# Super-Wide Impedance Bandwidth Planar Antenna for Microwave and Millimeter-Wave Applications

**DOI:** 10.3390/s19102306

**Published:** 2019-05-19

**Authors:** Mohammad Alibakhshikenari, Bal Singh Virdee, Chan H. See, Raed A. Abd-Alhameed, Francisco Falcone, Ernesto Limiti

**Affiliations:** 1 Electronic Engineering Department, University of Rome “Tor Vergata”, Via del Politecnico 1, 00133 Roma, Italy; alibakhshikenari@ing.uniroma2.it (M.A.); limiti@ing.uniroma2.it (E.L.); 2 Center for Communications Technology, School of Computing & Digital Media, London Metropolitan University, Center for Communications Technology, London N7 8DB, UK; b.virdee@londonmet.ac.uk; 3 School of Engineering & the Built Environment, Edinburgh Napier University, 10 Colinton Rd., Edinburgh EH10 5DT, UK; C.See@napier.ac.uk; 4 School of Engineering, University of Bolton, Deane Road, Bolton BL3 5AB, UK; 5 Faculty of Engineering & Informatics, University of Bradford, Bradford, West Yorkshire BD7 1DP, UK; R.A.A.Abd@Bradford.ac.uk; 6 Electric and Electronic Engineering Department, Universidad Pública de Navarra, 31006 Pamplona, Spain

**Keywords:** array antenna, microstrip patch antenna (MPA), slot antenna, simplified composite right/left-handed metamaterial (SCRLH MTM), multiple-output multiple-input (MIMO), radar, radio frequency identification (RFID) systems, millimeter-wave band

## Abstract

A feasibility study of a novel configuration for a super-wide impedance planar antenna is presented based on a 2 × 2 microstrip patch antenna (MPA) using CST Microwave Studio. The antenna comprises a symmetrical arrangement of four-square patches that are interconnected to each other with cross-shaped high impedance microstrip lines. The antenna array is excited through a single feedline connected to one of the patches. The proposed antenna array configuration overcomes the main drawback of conventional MPA with a narrow bandwidth that is typically <5%. The antenna exhibits a super-wide frequency bandwidth from 20 GHz to 120 GHz for S11 < −15 dB, which corresponds to a fractional bandwidth of 142.85%. The antenna’s performance of bandwidth, impedance match, and radiation gain were enhanced by etching slots on the patches. With the inclusion of the slot, the maximum radiation gain and efficiency of the MPA increased to 15.11 dBi and 85.79% at 80 GHz, which showed an improvement of 2.58 dBi and 12.54%, respectively. The dimension of each patch antenna was 4.3 × 5.3 mm^2^. The results showed that the proposed MPA is useful for various existing and emerging communication systems such as ultra-wideband (UWB) communications, RFID systems, massive multiple-output multiple-input (MIMO) for 5G, and radar systems.

## 1. Introduction

Demand for antennas that possess desirable characteristics such as light weight, low profile, and high gain have burgeoned significantly with the rapid development of modern wireless communication systems [1,2]. Antennas implemented on a microstrip medium exhibit some of these desirable properties, which makes them very popular in RF/microwave transceiver systems as they are compatible with integrated circuit technology and are relatively cheap and easy to fabricate [3,4,5,6,7,8,9,10]. In addition, microstrip patch antennas (MPAs) can be made to conform to planar and non-planar surfaces. The radiation mechanism arises from discontinuities at each truncated edge of the microstrip transmission line. The radiation at the edges causes the antenna to act slightly larger electrically than its physical dimensions, so in order for the antenna to be resonant, a length of microstrip transmission line slightly shorter than one-half a wavelength at the frequency is used. Various techniques have been previously developed to enhance the antenna’s impedance bandwidth and reduce its physical footprint, hence the MPA has become extensively used in various wireless communication applications. Nevertheless, conventional microstrip patch antennas still suffer from a narrow impedance bandwidth that is typically less than 5% with low radiation efficiency [1,2,3,4]. In addition, the operation of MPA is restricted to the microwave band.

In this paper, we propose a simple method to overcome the main drawback of the conventional microstrip patch antenna, and thereby realized a super-wide impedance bandwidth antenna. The design of the antenna was based on implementing four interconnected square patches in close proximity and arranged in an array configuration. Each patch constituting the antenna was loaded with a rectangular slot to improve its performance without increasing the size of the patches. This was implemented by simply etching a slot inside each radiating patch. The slot acts essentially like a series of left-handed capacitance and the resulting patch exhibits simplified composite right/left-handed (SCRLH) metamaterial properties [11,12,13]. The proposed microstrip patch antenna design is applicable for various existing and emerging communication systems such as ultra-wideband (UWB) communications, RFID systems, massive multiple-output multiple-input (MIMO) for 5G, and radar systems.

## 2. Proposed Microstrip Antenna Structure

**Figure 1:**
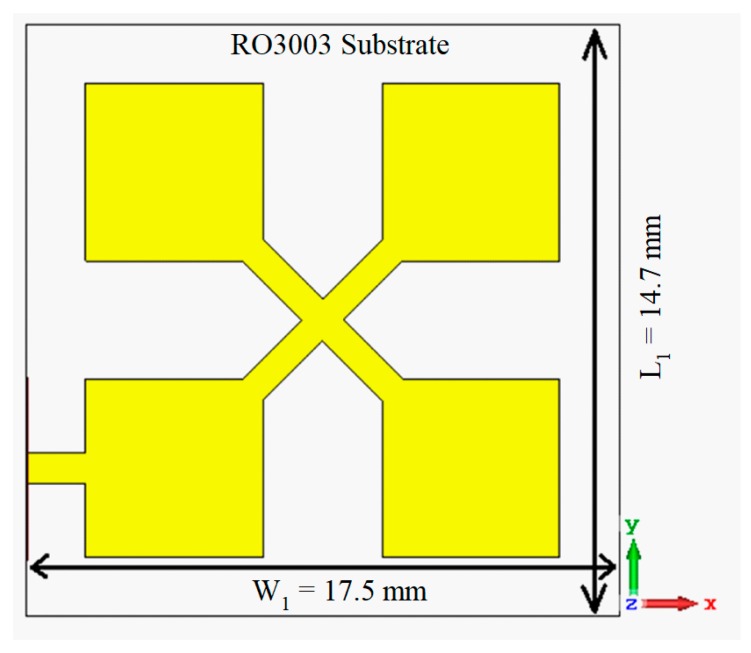
The proposed microstrip antenna array.

The proposed antenna structure was composed of four-square patches in a 2 × 2 arrangement, as shown in Figure 1. The antennas were interconnected with a cross-shaped high-impedance line. The design of the square patches was based on conventional theory. The width and length of the patch were calculated using the following standard design equations [14].

(1) $$Width=\frac{c}{2f_{o}\sqrt{\frac{\varepsilon_{r}+1}{2}}}$$

(2) $$Length=\frac{c}{2f_{o}\sqrt{\varepsilon_{eff}}}-0.824h\left[\frac{\left(\varepsilon_{eff}+0.3\right)\left(\frac{W}{h}+0.264\right)}{\left(\varepsilon_{eff}-0.258\right)\left(\frac{W}{h}+0.8\right)}\right]$$

(3) $$\varepsilon_{eff}=\frac{\varepsilon_{r}+1}{2}+\frac{\varepsilon_{r}-1}{2}\left[\frac{1}{1+12\sqrt{\frac{h}{W}}}\right]$$

The microstrip patch was designed at 20 GHz on standard theory on a high frequency ceramic-filled PTFE composite dielectric substrate by Rogers RO3003 with a dielectric constant of 3.0, loss-tangent of 0.001, and thickness of 0.13 mm. The physical dimensions of the proposed antenna configuration are given in Table 1. The resulting antenna had a low profile and was simple to design and fabricate. Unlike conventional microstrip antenna arrays, the proposed antenna array was excited through a single feedline connected to one of the antennas.

**Figure 2:**
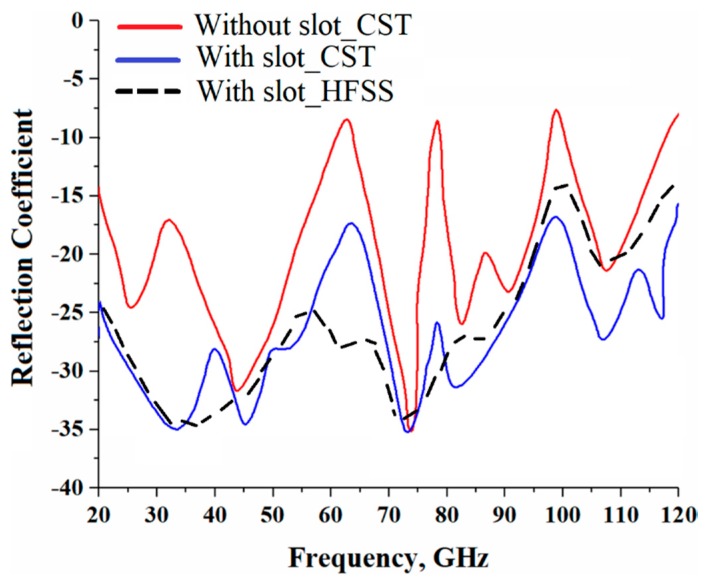
Reflection-coefficient (S_11_ < −10 dB) response of the microstrip antenna array “without” slots and “with” slots using two different commercially available 3D full wave electromagnetics simulation tools (CST Microwave Studio^®^ and HFSS™).

The reflection-coefficient response in Figure 2 of the proposed MPA array structure shows that its impedance bandwidth extended from 20 GHz to 120 GHz for S11 < −10 dB with four narrow band-notches at 62.5, 77.5, 97.5, and 120 GHz.

**Figure 3:**
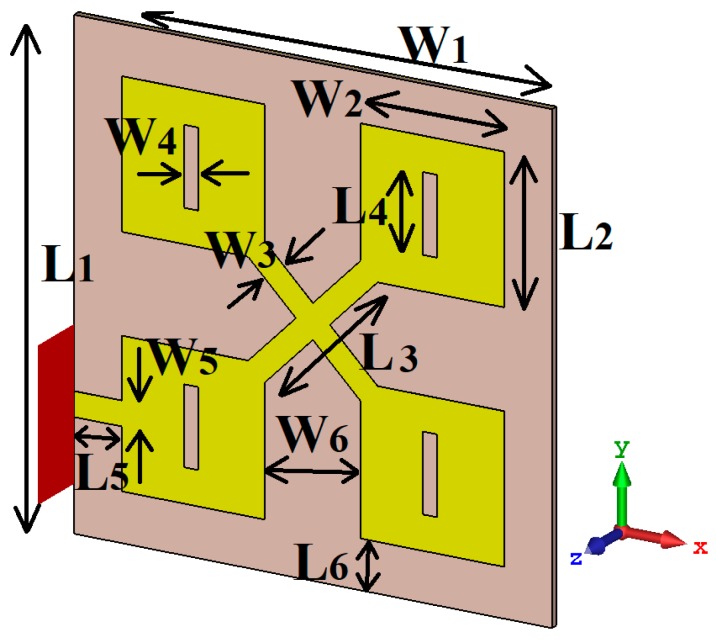
Configuration of the proposed microstrip antenna array with a ground-plane.

To improve the array’s performances and extend its effective aperture area, the four patches were loaded with a rectangular slot, as shown in Figure 3. With the addition of the slots, the reflection-coefficient was significantly improved. Now, an impedance bandwidth from 20 GHz to 120 GHz was achieved for S11 < −17.5 dB with no narrow band-notches. In the patch structure, the slot essentially acts like a series of left-handed capacitance and the resulting patch exhibited simplified composite right/left-handed (SCRLH) metamaterial properties [11,12,13]. It is evident from Figure 2 that there was a distinct improvement in the reflection-coefficient from 20–120 GHz. The improvement in the antenna’s performance can be attributed to a combination of the metamaterial effects and the complex interaction resulting from the surface currents over the antenna and electromagnetic fields. With the proposed technique, the dimensions of the antenna structure remained unaffected. It was, however, necessary to optimize the dimensions of the slots to enhance the reflection-coefficient response of the antenna array, and the optimized dimensions are given in Table 1.

**Figure 4:**
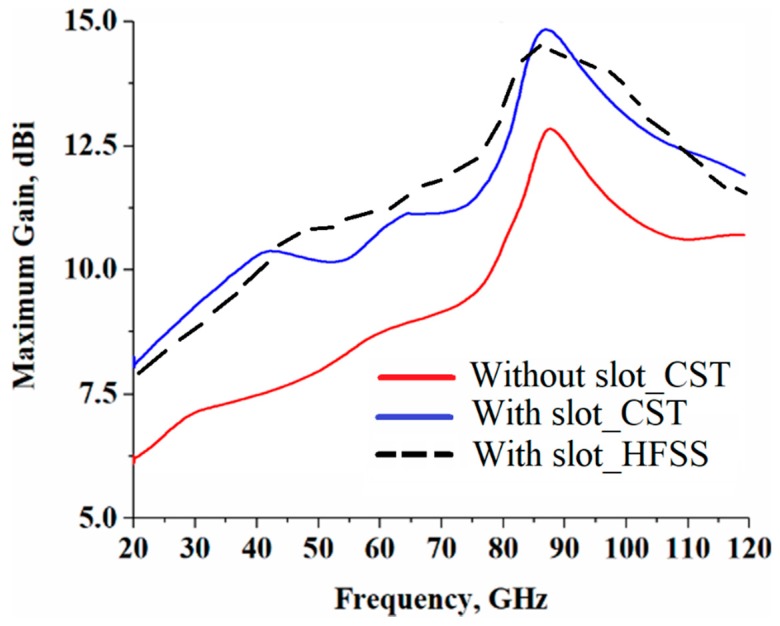
Gain response for both cases “with no” slots and “with” slots using two different commercially available 3D full wave electromagnetics simulation tools (CST Microwave Studio^®^ and HFSS™).

**Figure 5:**
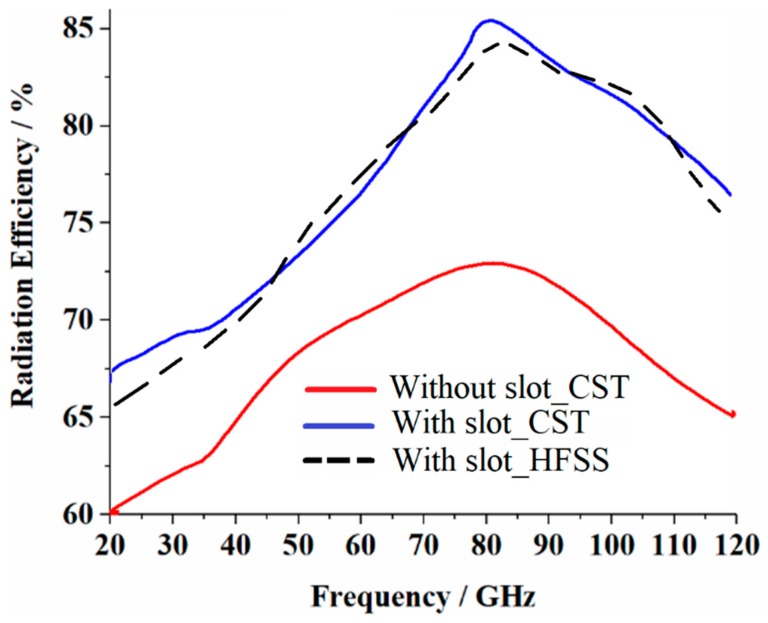
Radiation efficiency response for both cases “before applying” the slots and “after applying” the slots using two different commercially available 3D full wave electromagnetics simulation tools (CST Microwave Studio^®^ and HFSS™).

The radiation gain and efficiency of the antenna array with no slots and with slots are shown in Figure 4 and Figure 5, respectively. These figures show that with no slots, the antenna gain and efficiency reached a peak of around 12.53 dBi and 73.25% at 80 GHz, respectively, however, with the application of the slot, the optimum gain and efficiency improved to 15.11 dBi and 85.79% at 80 GHz, respectively. Therefore, an average improvement of 2.58 dBi and 12.54% in the maximum radiation gain and efficiency were achieved, respectively. The details of the radiation properties are presented in Table 2.

**Figure 6:**
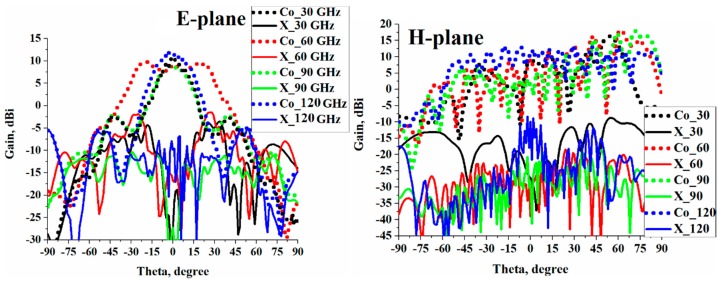
Co- and cross-radiation patterns of the proposed microstrip antenna arrays “with” slots in the E- and H-planes at spot frequencies over its operating band.

Co- and cross (X) polarization radiation patterns of the proposed microstrip antenna array in the E- and H-planes are shown in Figure 6 at spot frequencies of 30, 60, 90, and 120 GHz in its operating range. This shows that the antenna was directional in the E-plane with sidebands at about 15 dB down from the main beam. It was observed that at 60 GHz, the beamwidth doubled and the gain dropped by an average of 3 dB. In the H-plane, the beamwidth extended from around −50 to +80 degrees and the radiation gain varied with frequency. In both planes, the cross polarization was significantly below the main beam.

**Figure 7:**
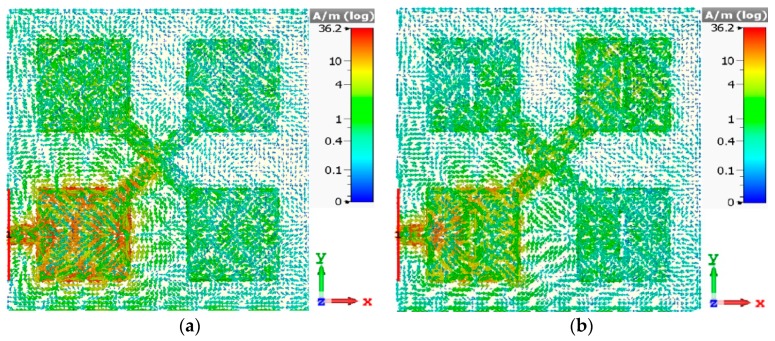
Surface current distributions at the spot frequency of 80 GHz, (**a**) “without” slots, and (**b**) “with” slots.

The surface current distributions before and after applying the slots at an arbitrary frequency of 80 GHz in the antenna’s operating range are shown in Figure 7. This figure shows that with no slots, the current was mainly concentrated around the excitation patch, however, when slots were introduced, the current was more evenly distributed between the four patches. This reveals that greater interaction was realized between the four patches, which resulted in a significantly improved reflection-coefficient over a super-wide frequency range.

It is worth commenting that to validate the results, we modeled and simulated the proposed structure with two different 3D full-wave electromagnetic simulation tools (CST Microwave Studio^®^ and HFSS™). There was excellent correlation between the CST Microwave Studio^®^ and HFSS™ results. CST Microwave Studio^®^ uses method of moments (MoM) to arrive at the solution whereas HFSS™ uses the finite element method (FEM).

## 3. Comparison with Other Recent Designs

The proposed antenna was compared to the planar wideband antennas reported to date in terms of design technique, size, dielectric constant, and operating frequency. The comparison is summarized in Table 3. Compared to other antennas, the proposed antenna has a much smaller footprint and operates over a significantly wider impedance bandwidth. In addition, it is simple to design and implement.

## 4. Conclusions

The feasibility of a novel configuration for a 2 × 2 microstrip patch antenna based on the metamaterial concept using CST Microwave Studio was shown to exhibit a super-wide impedance bandwidth, extending from 20 GHz to 120 GHz for S11 < −15 dB, which corresponded to a fractional bandwidth of 142.85%. The average gain and radiation efficiency of the antenna were 12 dBi and 78%, respectively, which showed a 4.0 dBi and 12% improvement after applying the slots. The proposed antenna structure overcomes the narrow bandwidth of conventional microstrip patch designs. The antenna can be used at microwaves and millimeter-wave applications including UWB, RFID systems, massive MIMO for 5G, and radar systems.

## Figures and Tables

**Table 1:** Antenna structural parameters.

L_1_	14.7 mm	W_1_	17.5 mm
L_2_	4.3 mm	W_2_	5.3 mm
L_3_	4.5 mm (λ_0_/4)	W_3_	0.3 mm (50 Ω)
L_4_	4.3 mm (0.52 × L_2_)	W_4_	0.52 mm (0.1 × W_2_)
L_5_	2.4 mm (λ_0_/4)	W_5_	0.3 mm (50 Ω)
L_6_	2.4 mm (λ_0_/4)	W_6_	0.32 mm (0.6 × W_2_)

**Table 2:** Radiation performance parameters.

**Radiation Gain (with No Slot)**		
Minimum	Maximum	Average
5.75 dBi	12.53 dBi	8 dBi
**Radiation Gain (with Slot)**		
7.88 dBi	15.11 dBi	12 dBi
**Improvement**		
2.13 dBi	2.58 dBi	4 dBi
**Radiation Efficiency (with No Slot)**		
Minimum	Maximum	Average
60.82%	73.25%	66%
**Radiation Efficiency (with Slot)**		
67.41%	85.79%	78%
**Improvement**		
6.95%	12.54%	12%

**Table 3:** Comparison with recently reported antennas.

Refs.	Technique	Antenna Size (mm^3^)	Dielectric Constant	Operating Frequency (GHz)
[15]	CPW-fed antenna	24 × 30.5 × 1.5	3.38	3.1–10.6
[16]	Inverted L-resonator & circular slotted GND	40 × 30 × 1.2	4.4	3.1–10.6
[17]	Annular slot	26 × 24 × 1.6	4.6	3–10.6
[18]	Rectangular slots	16 × 14 × 1	4.4	3.2–10
[19]	Circular slots	30 × 26 × 1.6	4.4	2.5–11
[20]	Inverted U-strip	45 × 50 × 1.27	6.0	3.1–10.6
[21]	Split ring resonators	30 × 26 × 1.6	3.5	2.4–10.1
[22]	lamp shaped antenna	28×15× 1.6	4.4	2.7–14
[23]	Cap. Integrated antenna	30.5 × 24 × 1.5	3.3	3.1–10.6
[24]	L-shaped stub	46 × 42 × 1	4.4	3.1–10.6
[25]	Loading quarter wavelength resonating strip	38 × 30 × 1.6	4.4	3.1–10.6 and 2.4–2.5
[26]	Loading TL-MTM within UWB antenna	38.5 × 46.4 × 1.6	4.4	3.1–10.6 and 2.43–2.49
[27]	Half elliptical ring with a U-shaped slot	32 × 32.6 × 1.6	4.4	3.1–10.6
[28]	Loading quarter wavelength resonating strip at the center of the patch	50 × 24 × 1.6	4.4	3.1–11.4 and 2.18–2.59
[29]	Loading parasitic strip	46 × 20 × 1.0	2.4	3.1–10.6 and 2.40–2.48
[30]	Loading quarter wavelength resonating strip at the center of the patch	42 × 24 × 1.6	4.4	3.1–12.0 and 2.30–2.50
[31]	Loading strip-line to the patch	45 × 32 × 1.0	4.4	3.1–10.6 and 2.40–2.50
[32]	Capacitors loaded miniaturized resonator in the ground plane	30 × 31 × 1.5	3.38	3.1–10.6 and 2.4–2.48
[33]	Band-pass filter integration with combination of GCPW, grounded reflector, and CPW feed line	35 × 24.4 × 2	3.38	2.8–6
[34]	Dielectric loading	61 × 61 × 8	~4.0	1.6–12
**This paper**	**SCRLH metamaterial**	**14.7 × 17.5 × 0.13**	**3.0**	**20–120**
